# Water Molecule(s) Inside the Selectivity Filter of Aquaporin 1: A DFT Study

**DOI:** 10.3390/molecules31030433

**Published:** 2026-01-27

**Authors:** Silvia Angelova, Luis Manuel Frutos, Nikoleta Kircheva, Yulian Zagranyarski, Obis D. Castaño, Todor Dudev

**Affiliations:** 1 Institute of Optical Materials and Technologies “Acad. J. Malinowski”, Bulgarian Academy of Sciences, 1113 Sofia, Bulgaria; sea@iomt.bas.bg (S.A.); nkircheva@iomt.bas.bg (N.K.); 2 Departamento de Química Analítica, Química Física e Ingeniería Química, Universidad de Alcalá, 28805 Alcalá de Henares, Madrid, Spain; luisma.frutos@uah.es (L.M.F.); obisd.castano@uah.es (O.D.C.); 3 Instituto de Investigación Química “Andrés M. del Río” (IQAR), Universidad de Alcalá, 28805 Alcalá de Henares, Madrid, Spain; 4 Faculty of Chemistry and Pharmacy, Sofia University “St. Kliment Ohridski”, 1164 Sofia, Bulgaria; ohjz@chem.uni-sofia.bg

**Keywords:** aquaporin, water channel, selectivity, mechanical stress, nano-Newton force

## Abstract

Aquaporin 1 (AQP1) is a transmembrane protein that acts as a highly selective channel for the rapid passage of water across cell membranes, driven by osmotic gradients. The narrowest part of the water channel pore—the selectivity filter (SF)—plays a key role in ensuring selective and efficient water transport. In this study, density functional theory (DFT) at the M062X/6-311+G(d,p) level was used to identify the preferred position of the water molecule(s) inside the SF and to elucidate the forces that lead to its displacement during permeation. A systematic scan along the pore axis identified a well-defined energy minimum where a single water molecule was optimally stabilized by hydrogen bonds with SF residues. A second water molecule was introduced to study how the incoming water affects the translocation of the first water molecule. The resulting energy and force profiles reveal that the approaching water molecule gradually pushes the bound water forward, ultimately occupying its favorable binding site. These results provide an atomistic description of the positioning and displacement of water molecules in SF and offer a quantitative view of the fundamental interactions that govern water transport in AQPs.

## 1. Introduction

Water, which covers about two-thirds of Earth’s surface and is necessary for sustaining life on our planet, is known for its phenomenal properties as compared to other substances [1,2]. Since the pioneering work of L. J. Henderson in 1913 [3], scientists have been striving to understand the relationship between the unusual characteristics of water and the existence of life. Water’s remarkable physicochemical properties—including its high heat capacity, strong hydrogen bonding, exceptional solvent abilities, and anomalous density profile—form the very foundation of biological organization in living systems [1,4]. These unique features support macromolecular stability, enable biochemical reactions, and facilitate the transport of metabolites and ions, making water indispensable in all known domains of life [5,6].

At the cellular level, the regulated movement of water across biological membranes is essential for maintaining osmotic balance, cell volume, and overall physiological homeostasis [7,8]. Biological membranes, composed principally of phospholipid bilayers and embedded proteins, act as selectively permeable barriers [9]. Although limited passive diffusion of water can occur through the lipid bilayer, this mechanism is insufficient to explain the exceptionally rapid and tightly controlled water flux observed in specialized tissues such as renal tubules, red blood cells, secretory glands, and plant roots. These discrepancies have led to the long-standing hypothesis suggesting that, in order to facilitate high-throughput permeability without compromising membrane integrity or ion gradients, special water channels must exist [10,11,12,13].

The identification of water channel proteins, aquaporins (AQPs), has addressed the century-long mystery of facilitated water movement across biological membranes [14,15]. In 1985, following a decade of systematic studies on water transport in human red blood cells (RBCs), the first water channel protein, now known as aquaporin-1 (AQP1), was discovered by Benga’s group [16]. AQP1 was detected in situ in human RBC membranes using selective radiolabeling with the water-transport inhibitor [^203^Hg]-PCMBS [17,18]. The labeled polypeptide appeared in the 35–60 kDa region on electrophoretic profiles, and the findings were published in 1986 [16]. Independently, Agre’s group later purified a 28 kDa RBC membrane protein, termed CHIP28, and in 1992 demonstrated that it functions as a selective water channel [14,19]. CHIP28 was subsequently recognized as the same class of protein and is now known as AQP1. AQP1 is widely expressed across mammalian tissues where efficient water transport is essential and provides definitive evidence of the molecular machinery responsible for rapid transmembrane water movement [20,21]. Physiologically, it is expressed at very high levels in renal proximal tubule cells, where it localizes to both apical and basolateral membranes, as well as in the endothelial lining of capillaries, plants, and animals [9]. This channel contributes to several key physiological processes, including regulation of body fluid balance, control of endothelial water permeability, production of cerebrospinal fluid, and promotion of cell migration [22]. It also plays an essential role in the counter-current exchange system that regulates urine concentration. Altered AQP1 expression or function has been linked to a broad spectrum of disorders, such as edema, various pulmonary pathologies, glaucoma, and tumor progression [23]. Recent findings additionally point to the role of AQP1 in angiogenesis, tissue repair, organ regeneration, and oncogenic processes [24].

Today, it is well established that AQPs are integral membrane proteins that form narrow, highly selective pores, allowing water to traverse biological membranes at rates several orders of magnitude higher than simple diffusion [22]. Since the discovery of AQP1, more than 1700 AQP sequences have been identified across all domains of life, including bacteria, plants, and animals. Over the years, members of the AQP family have been shown to play key roles in numerous physiological processes and have been implicated in the pathophysiology of a wide range of clinical disorders [25,26,27,28]. Functionally, AQPs are generally classified into two major groups [9]: classical (orthodox) aquaporins, homologues that conduct water exclusively, and aquaglyceroporins (GLPs), homologues permeable to both water and small neutral solutes, primarily glycerol.

**Figure 1:**
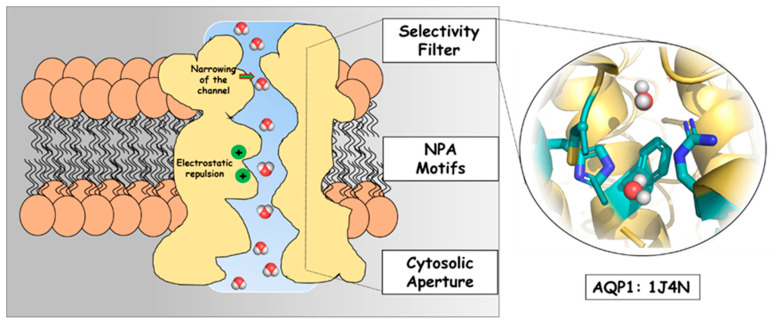
A simple model of the water pore in AQP1 (PDB: 1J4N), with a focus on the selectivity filter as deposited in the protein data bank. The numbering of amino acids lining the selectivity filter is further given in Section 3.1. Models Used.

The elucidation of AQPs’ structure and function revolutionized the understanding of membrane physiology [21,29,30]. Herewith, a simple presentation of the main components of the water pore in the AQP1 water channel (PDB number: 1J4N) is demonstrated in Figure 1. Dumb-bell-like in shape, it contains the following general elements after the extracellular vestibule: an extended narrow pore or selectivity filter (SF), where the channel narrows substantially (constriction region), the so-called NPA motifs, and a cytoplasmic vestibule. The conserved architecture, based on dual NPA (Asparagine–Proline–Alanine) motifs and a distinctive aromatic/arginine (ar/R) selectivity filter (SF, the narrowest part of the pore), was identified by high-resolution structural analyses [9,31]. All these features guarantee strict water selectivity while excluding cationic species, and protons in particular. It is reasonable to infer that all AQPs function via a generally similar transport mechanism since they have a conserved overall architecture and show strong sequence similarity in their pore-forming regions.

When elucidating the mechanistic details of water transport through aquaporins, a multifaceted approach must be utilized, including computational studies providing a crucial insight into the processes at a molecular level. Molecular dynamics (MDs) simulations have made it possible to quantify permeation rates, visualize water permeation at atomic resolution, and analyze the dynamic behavior of important structural elements like the dual NPA motifs and the aromatic/arginine (ar/R) selectivity filter [32,33]. These simulations have shed light on the energetic barriers preventing proton transport through the pore, hydrogen-bond network rearrangements, and single-file water movement. On the other hand, the electronic structure contributions to water selectivity, the impact of conserved residues on the channel’s electrostatic landscape, and the mechanisms underlying proton exclusion have all been made clearer by quantum mechanical techniques like density functional theory (DFT) and QM/MM hybrid methods [34,35,36,37]. Taken together, these computational methods have created a complete mechanistic framework for understanding how AQP works by combining structural data from X-ray crystallography with dynamic water transport phenomena. The obtained new information can then be used to design biomimetic channels and possible therapeutic modulators of AQP activity.

Beyond thermal fluctuations, biomolecules are often subject to mechanical stresses that may alter local interactions and binding preferences. In this regard, force analysis (either computed directly or inferred from energy gradients along a reaction coordinate) provides a direct mechanistic link between structure and function. For instance, it has been shown that directional nano-Newton forces can modulate the metal affinity and selectivity of metal binding sites in proteins [38].

Research on AQP1 continues to yield deep insights into fundamental cellular processes, as well as the mechanisms underlying human disease. In this context, the current work aims to clarify the structural and electronic features that govern water coordination and selectivity within the narrowest region in the AQP1 pore from the selectivity filter, as presented in Figure 1. By applying DFT methods to a simplified model of water–protein architecture mimicking the amino-acid composition in the constriction region, this work seeks to refine the molecular understanding of the fundamental interactions that govern water transport through AQPs.

## 2. Results

### 2.1. Single Water Molecule Inside the Pore

**Figure 2:**
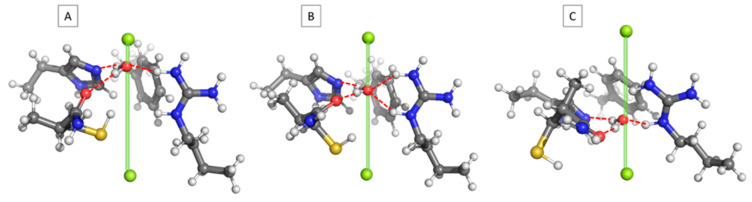
Optimized structures of the complexes between the AQP1 SF and a water molecule inside the pore, located at (**A**) 1.5 Å, (**B**) 3.0 Å, and (**C**) 5.0 Å from the Bq1 atom.

**Figure 3:**
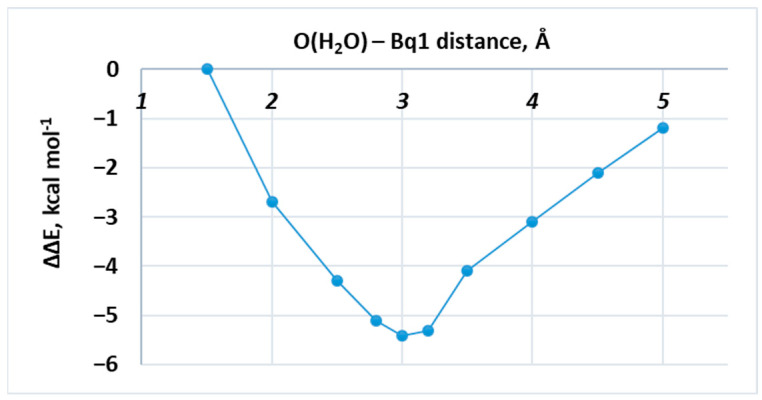
Graph of the change in the relative interaction energies ΔΔE with respect to the distance between the oxygen atom from the single water molecule to the ghost atom Bq1 (O(H_2_O)–Bq1) in Å.

The interaction between the AQP1 SF and a single water molecule placed at different locations along the pore axis was evaluated. Figure 2 gives snapshots of three representative structures: the water molecule placed near the entrance of the SF (O(H_2_O)–Bq1 distance = 1.5 Å; Figure 2A), the water molecule located at the central part of the aperture (O(H_2_O)–Bq1 distance = 3.0 Å; Figure 2B), and the water molecule close to the exit of the SF (O(H_2_O)–Bq1 distance = 5.0 Å; Figure 2C). As can be seen, in all cases, the passing water molecule forms hydrogen bonds with the attractor amino acids, although with varying strength. These are as follows: His182 with hydrogen bonds between N(His) and H(H_2_O) ranging from 1.80 to 1.78 and 1.69 Å for structures 2A, 2B, and 2C, respectively; Cys191(backbone carbonyl), where the O(C=O)–H(H_2_O) bond length fluctuates between 2.00 (Figure 2A) and 1.83 (Figure 2B)/1.83 Å (Figure 2C); and Arg197 whose NH groups are engaged in hydrogen bond formation with the oxygen atom of the water molecule. It is interesting to note that in the latter case, the two NH fragments do not participate equally in creating the hydrogen bonds, which depend on the particular position of the passing water molecule. Thus, in the early structure (Figure 2A), the water molecule interacts with the upper NH group only (H(NH)–O(H_2_O) bond length = 1.71 Å), whereas the lower NH fragment is virtually excluded from interacting with water (H(NH)–O(H_2_O) bond length = 3.12 Å). In the next structure (Figure 2B), the two NH groups participate almost equally in hydrogen bond formation and are characterized by bond lengths of 1.85 (upper fragment) and 1.94 Å (lower unit). In the third case (Figure 2C), the interaction with the upper entity weakens (hydrogen bond length of 2.00 Å), whereas that with the lower NH fragment strengthens (hydrogen bond length = 1.73 Å). These structural changes are reflected by the respective energies of interaction. As shown in Table 1 and Figure 3, the relative interaction energy decreases (meaning more favorable) when the water molecule descends from O(H_2_O)–Bq1 of 1.5 Å, and, after reaching the minimum at O(H_2_O)–Bq1 of 3.0 Å, takes an upward turn (less favorable) and gradually increases toward O(H_2_O)–Bq1 = 5.0 Å. Note that the energy minimum corresponds to the most stable structure, where the water molecule is engaged in forming four hydrogen bonds, unlike other structures, where the number of hydrogen bonds is three. It is not meant, however, to represent the full free-energy barrier governing transport through the channel, but rather provides a thermodynamic ‘snapshot ‘of the first constriction region in the SF interacting with a water molecule in the most energetically favorable fashion. The addition of a second H_2_O molecule provides some further insight into the water transport mechanism inside the applied SF model, but again in terms of the mechanochemical framework and not from a kinetic point of view.

### 2.2. The Second Water Molecule Repels the First One

**Figure 4:**
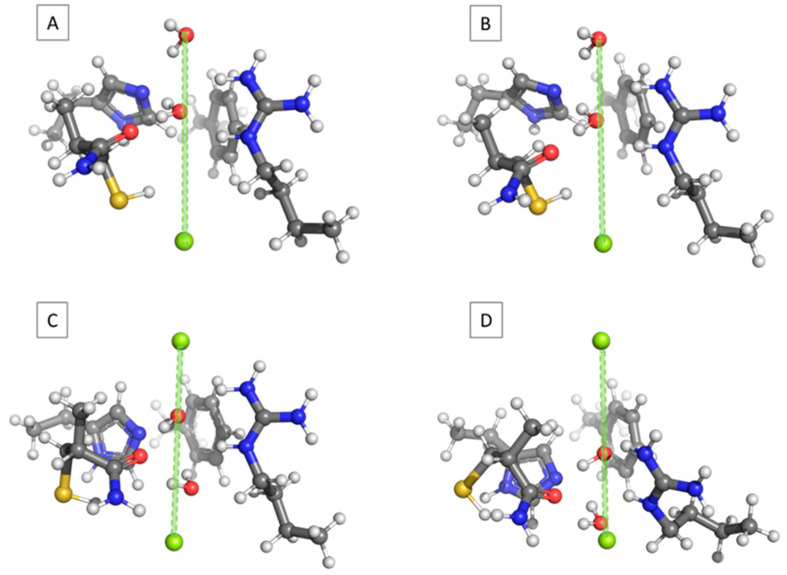
Complexes between AQP1 SF and the two water molecules inside the pore. (**A**) The initial structure with the first water molecule optimally placed, with respect to the amino acid residues lining the pore (O(H_2_O)–Bq1 = 3.0 Å), and the attacking water molecule at the entrance of the pore (O(H_2_O)–Bq1 = 0.0 Å); (**B**) the final (optimized) structure of (**A**), where the first water molecule has moved to O(H_2_O)–Bq1 = 3.17 Å; (**C**) the optimized structure of the complex with the attacking Wat2 frozen at O(H_2_O)–Bq1 = 3.0 Å and Wat1 pushed down 3.03 Å, with respect to the attacker; and (**D**) the optimized structure of the complex with the second water molecule frozen at O(H_2_O)–Bq1 = 4.5 Å and the first one pushed down 2.98 Å, with respect to the attacker.

The next batch of calculations started with a non-frozen water molecule (Wat1), optimally placed in the central part of the channel aperture (the most energetically favorable structure at O(H_2_O)–Bq1 of 3.0 Å; see above) and being subjected to the influence of another water molecule (Wat2) coming down from the pore entrance (Figure 4A). Note that, in accordance with the experimental observations [21], water molecules are modeled in a manner that prevents them from forming a hydrogen-bonded chain/dimer. For example, in the structure depicted in Figure 4A, the attacking water molecule Wat2 (with its oxygen atom frozen during the optimization) was placed at 3.0 Å from Wat1, which, on its side, was allowed to fully relax during the optimization process. At the end of the optimization procedure, Wat1 was found not to be attracted to the attacker but pushed down to 3.17 Å, with respect to Bq1 (Figure 4B). For each consecutive step, the optimized position of Wat1 during the previous optimization was taken as a starting point for the next optimization, where Wat2 was moved downward with another increment and kept frozen. In all the cases, the attacking Wat2 repels Wat1 further down the pore. The optimized distance between the two water molecules for the entire set of calculations falls in the range of about 3.0 Å (Table 2). When considering the hydrogen bond formation, it should be noted that this type of interaction is observed only between the amino acid residues and the single H_2_O molecules, but none exists between the two waters in any of the optimized constructs. Overall, Wat2 displaces Wat1 in a gradual manner by occupying its former positions in the pore aperture (Figure 4C). The interaction energy within the complex decreases until the attacking particle reaches the most favorable position at O(Wat2)–Bq1= 3.0 Å. After that, the relative interaction energy, ∆∆E, starts increasing due to less benign interactions between the water molecules and the pore wall (Figure 4D). The repulsive forces experienced by the O(Wat1) under the influence of Wat2 vary between 0.44 and 1.52 nN and depend on a multitude of factors, including the specific interactions between the selectivity filter and the passing water molecules and, mostly, those between Wat1 and Wat2 themselves. Note that the strongest impact on Wat1 is detected for those structures where the O–O distance is below 3.0 Å (Table 2).

In general, the presented computational study delineates the importance of the protein surroundings in the selectivity filter. It shows explicitly how the interaction energy between a water molecule and the amino acid residues in the constriction region changes with the distance from the ‘entrance’ (see Table 1 and Figure 3). It should be noted that the most energetically favorable position of this single H_2_O corresponds to the experimentally reported data [21]. Such water-binding nodes are found at several positions in the otherwise hydrophobic filter. Their aim is to reduce the energy barrier to water transport, while their low number minimizes the solute–pore interaction. When analyzing the PDB structure 1J4N, we found that only four waters were positioned throughout the whole selectivity filter (and only two in the modeled constriction region), namely at these nodes, as expected. Furthermore, the probable mechanism of water transport through this region was modelled, as well. It was found that the H_2_O molecules do not form a contiguous hydrogen-bonded chain, as the ‘attacking’ water rather repels the other one down the pore by applying force on it, which increases substantially with the distance between Wat1 and Wat2. A turning point for this interaction was found to be the distance below 3 Å, where not only the force enhances, but the relative interaction energy becomes positive, e.g., unfavorable. These observations obtained by the presented DFT study fall completely in agreement with previously reported experimental findings regarding the probable water transport in the selectivity filter of AQP1. Nevertheless, the results provide only a partial insight into this intriguing matter, being drawn from a thermodynamic point of view. The kinetic component could be further addressed by other computational tools, such as molecular dynamics simulations and/or QM/MM methods, which fall out of the scope of the presented study.

## 3. Materials and Methods

### 3.1. Models Used

**Figure 5:**
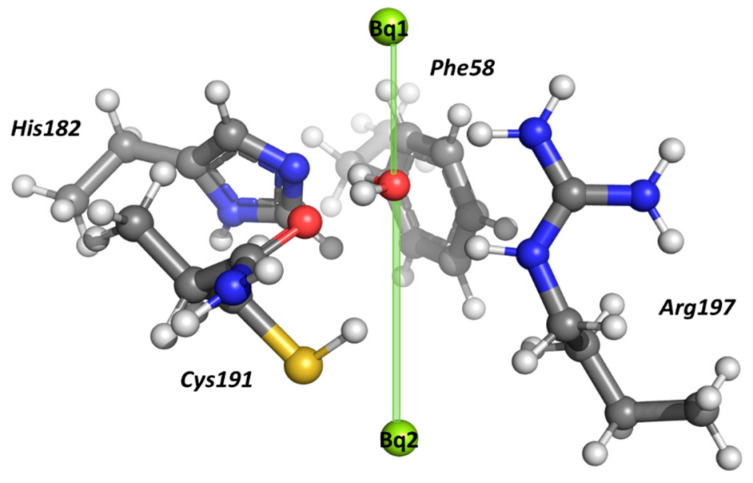
The selectivity filter of AQP1, pore axis (formed by the ghost atoms Bq1 and Bq2), and a water molecule inside the pore.

The interactions of the water molecule(s) inside the selectivity filter of human AQP1 were evaluated. For this purpose, the structure of the SF was modeled according to the experimental crystallographic data [21]. The constriction region comprises the side chains of His182, Arg197, and Phe58, and the backbone amide group of Cys191. All the side chains were modeled explicitly (Figure 5). The charge of the amino acid residues was modelled in accordance with their pKa values at the neutral pH of the surrounding medium, rendering arginine positively charged and histidine neutral [39,40]. The end Cα atom of His182, Arg197, and Phe58 was capped with a methyl group, whereas the backbone amide group of Cys191 was explicitly modeled, as it interacts with the passing water molecule [21]. All Cα atoms were kept frozen to their initial positions during the structure optimization in order to preserve the integrity of the pore. The rest of the protein atoms were allowed to fully relax and find their optimal orientation. The pore axis was marked by two “ghost atoms” (Bq1 and Bq2) spanning 8.0 Å. The reaction coordinate was set up as the distance between Bq1 and the oxygen atom of H_2_O (Figure 5). It should be noted that within the simplified model of the selective filter considered in the present model, structural experimental data indicate the presence of only two water molecules. The inclusion of additional water molecules would require an extended representation of the selective filter, incorporating more amino acid residues, which is beyond the scope of the current computational framework and would significantly increase the computational costs.

### 3.2. Computational Strategy

Two types of calculations were performed: In the first one, a single water molecule was modeled to move stepwise along the pore axis with an increment of 0.2–0.5 Å (downward direction from the extracellular to the cytoplasmic end of the channel). The distance between O(H_2_O) and Bq1 was frozen while the rest of the water parameters were fully optimized. The energy in the interaction between the SF and the water molecule was evaluated for each water position and used to map the energy profile. The most energetically favorable construct was located. With this data at hand, a second type of calculations was initiated focused on the effect of a second water molecule on the position/energetics of the first one. The second H_2_O was moved stepwise by 1.0–0.5 Å toward the first one, which was initially located at the most favorable position inside the SF (see above). The distance between the oxygen atom of the second (attacking) water molecule and Bq1 was kept frozen, whereas the first water molecule was allowed to fully relax and find its optimal position inside the pore. The energy of interaction between the SF and the two water molecules, as well as the magnitude of the repulsive forces imposed on the oxygen atom of the first H_2_O by the incoming second water molecule, were evaluated.

To quantify how the affinity of the selectivity filter for water changes along the insertion coordinate, we first computed the interaction energy at each position R, defined as the distance $R\equiv d[O(H_{2}O)-Bq1]$:

$$E_{int}(R)=E_{SF+H_{2}O}(R)-E_{SF}-E_{H_{2}O}$$

where $E_{SF+H_{2}O}(R)$ is the electronic energy of the SF–water complex optimized for the corresponding values of R, where $E_{SF}$, and $E_{H_{2}O}$ are the energies in the isolated fragments computed at the same level of theory. Therefore, the relative interaction energies are defined as:

$$\Delta \Delta E(R)=E_{int}(R)-E_{int}(R_{ref}),$$

using $R_{ref}=1.5$ Å as the reference configuration (thus ΔΔE=0 at $R_{ref}$, as indicated in Table 1). With this convention, ΔΔE(R)<0 indicates that the SF–water interaction is more stabilizing than at the reference position (i.e., a more favorable binding environment at that R), whereas ΔΔE(R)>0 denotes a less favorable interaction. Therefore, the ΔΔE(R) profile can be interpreted as an effective potential energy curve along the insertion coordinate as follows: minima identify preferred water locations, and the slope −d(ΔΔE)/dR is directly related to the effective force acting along R. This force–work interpretation follows mechanochemical frameworks in which energy variations along a well-defined path are expressed as the work developed by an effective force projected onto the corresponding reaction (or insertion) coordinate [41].

### 3.3. DFT Calculations

The Gaussian 16 suite of programs was employed [42]. The M062X method [43], in conjunction with a 6−311+G(d,p) basis set, was used in the calculations. The combination of this theoretical method and a Pople-style triple ζ-basis set was meticulously calibrated and validated in our previous studies, with respect to available experimental data, and was proven to be reliable, as it correctly reproduced the geometrical parameters and energies in a series of representative complexes [44,45]. However, taking the optimized geometries of the structures presented in Figure 2, additional single-point calculations at the M062X/6-311++G(d,p); B3LYP/6-311+G(d,p); B3LYP-D3/6-311+G(d,p); and ωb97x/6-311+G(d,p) were performed. The interaction between the AQP1 SF and a single water molecule placed at these three different locations along the pore axis was evaluated and presented in Table 3 to provide a comparison between the chosen M062X/6-311+G(d,p) level of theory and the other considered functionals. The results in parentheses correspond to calculated interaction energies corrected by the Boys and Bernardi procedure for basis set superposition error. Note that all the reported results stay in close proximity and within the error of the method. The BSSE correction, amounting to about 0.3 kcal mol^−1^, does not alter the general conclusions and the presented overall trends.

Polarizable continuum dielectric (PCM [46]) optimizations were performed in a low-dielectric medium (diethyl ether; ε = 4), as the selectivity filter of the aquaporin is known to have limited solvent accessibility [47] and water confined in narrow channels exhibits a reduced effective dielectric response relative to bulk water [48,49].

The forces acting on the oxygen atom of the first water molecule, under the influence of the approaching second one, were obtained at the stationary point of the respective construct.

## 4. Conclusions

Life on Earth is based on water and its transport throughout the cells of all living organisms. Since the discovery of aquaporin-1 (AQP1) by Benga’s group, this intriguing protein has been the main focus of a variety of scientific studies to investigate its structure, mechanism of water transport, and selectivity toward other small molecules. The presented herewith computational research aimed to contribute to this field by applying the tools of DFT methodology. By starting with modelling the first constriction region in the selectivity filter of the water pore, in accordance with the experimentally provided data, the most energetically favorable position of a single H_2_O in the surroundings of the amino acid residues was delineated. It was found to be at around 3 Å from the entrance of the SF, where the hydrogen bond formation between the solute and the protein environment is maximized. Moreover, a probable mechanism of water transport through the modelled region was demonstrated as follows: an ‘attacking’ H_2_O molecule was found to take the position of the previous water by repelling it downward through the pore. This outcome not only corresponds to the experimentally reported composition of the water molecules inside the pore of AQP1, but also guarantees the water selectivity of the filter. Although much has recently been revealed, and the presented study provides more evidence on the observed processes at a molecular level, quite intriguing questions still await their answers, such as how a mutation in the amino acid residues would affect the general conclusions so far, or if and how an artificial water channel could be designed to mimic the extraordinary features of the aquaporins.

## Figures and Tables

**Table 1:** The relative interaction energies (ΔΔE) for complexes between AQP1 SF and a single water molecule inside the pore located at different positions with respect to the Bq1 atom.

O(H_2_O)–Bq1 Distance (Å)	ΔΔE (kcal mol^−1^)
1.5	0
2.0	−2.7
2.5	−4.3
2.8	−5.1
3.0	−5.4
3.2	−5.3
3.5	−4.1
4.0	−3.1
4.5	−2.1
5.0	−1.2

**Table 2:** Forces on O(Wat1), optimized O(Wat1)–O(Wat2) distances, and relative interaction energies, ΔΔE, for the complexes between AQP1 SF and two passing water molecules.

O(Wat2)–Bq1 Distance (Å)	Force on O(Wat1) (nN)	Optimized O(Wat1)–O(Wat2) Distance (Å)	ΔΔE (kcal mol^−1^)
0.0	0.44	3.17	0.0
1.0	0.57	3.24	−1.70
1.5	0.54	3.21	−2.10
2.0	0.64	3.08	−3.47
2.5	1.14	3.07	−4.92
3.0	1.12	3.03	−5.15
3.5	1.29	2.96	−3.38
4.0	1.50	2.95	−0.63
4.5	1.52	2.98	2.39
5.0	1.29	2.94	4.52

**Table 3:** Relative interaction energies (ΔΔE) in kcal mol^−1^, with respect to the complex at 1.5 O(H_2_O)–Bq1 distance (Å) for complexes between AQP1 SF and the single water molecule inside the pore obtained through the application of different theoretical levels. The numbers in parentheses are BSSE corrected.

Method	ΔΔE (kcal mol^−1^) at 3 Å O(H_2_O)–Bq1 Distance (Å)	ΔΔE (kcal mol^−1^) at 5 Å O(H_2_O)–Bq1 Distance (Å)
M062X/6-311+G(d,p)	−5.4 (−5.0)	−1.2 (−0.9)
M062X/6-311++G(d,p)	−5.3 (−5.0)	−1.2 (−0.8)
B3LYP/6-311+G(d,p)	−4.7 (−4.4)	−0.2 (0.1)
B3LYP-D3/6-311+G(d,p)	−5.5 (−5.3)	−1.2 (−0.9)
ωb97x/6-311+G(d,p)	−5.9 (−5.6)	−2.0 (−1.7)

## Data Availability

The data presented in this study are available on request from the corresponding author.
